# Radiographic Sagittal Tibio-Talar Offset in Ankle Arthrodesis—Accuracy and Reliability of Measurements

**DOI:** 10.3390/jcm9030801

**Published:** 2020-03-16

**Authors:** Sophie Schieder, Elena Nemecek, Reinhard Schuh, Alexander Kolb, Reinhard Windhager, Madeleine Willegger

**Affiliations:** 1 Department of Orthopedics and Trauma Surgery, Division of Orthopedics, Medical University of Vienna, 1090 Vienna, Austria; schiedersophie@gmail.com (S.S.); elena.nemecek@meduniwien.ac.at (E.N.); reinhard.schuh@me.com (R.S.); alexander.kolb@meduniwien.ac.at (A.K.); reinhard.windhager@meduniwien.ac.at (R.W.); 2 Department of Trauma Surgery, Kepler University Hospital Linz, 4021 Linz, Austria

**Keywords:** reliability, sagittal alignment, ankle arthrodesis, tibio-talar offset

## Abstract

Radiographic outcome assessment of ankle arthrodesis (AA) requires accurate measurement techniques. This study aimed to identify the most reliable methods for sagittal tibio-talar alignment measurements with regard to the tibio-talar offset after AA. Lateral weight-bearing radiographs of 38 fused ankles were selected for retrospective review. The sagittal tibio-talar angle (STTA), the modified tibio-talar ratio (mT-T ratio) and the sagittal tibio-talar offset (tibCOR, procLAT) were measured by three independent observers. Intra- and interobserver correlation coefficients (ICC) and mean measurement differences were calculated to assess measurement reliability and accuracy. By defining the talar longitudinal axis as a line from the inferior aspect of the posterior tubercle of the talus to the most inferior aspect of the talar neck, STTA showed excellent (ICC 0.924; CI 95% 0.862–0.959) and mTT-ratio provided high (ICC 0.836; CI 95% 0.721–0.909) interobserver reliability, respectively. For tibio-talar offset measurement the tibCOR method showed superior reliability and better interobserver agreement compared to the procLAT technique. The STTA and a modified T-T ratio are recommended for future scientific radiographic measurements in AA.

## 1. Introduction

Multiplanar deformity in ankle arthritis is a common condition due to a high prevalence of posttraumatic arthritis [1,2]. Ankle arthrodesis (AA) is still a worldwide commonly used operative procedure in end-stage disease [3,4,5,6].

In order to create a plantigrade and well-aligned AA, the coronal, axial, and sagittal tibio-talar alignment has to be addressed. Sagittal plane deformities (i.e., pes equinus or pes calcaneus) alter joint reaction forces and biomechanical properties to a greater degree than coronal plane deformities (i.e., varus and valgus alignment), because the subtalar joint compensates for coronal tibio-talar malalignment to a certain degree [7,8,9,10]. Furthermore, the sagittal position of the talus relative to the tibial axis (tibio-talar offset) has to be taken into account. A posteriorly located talus shortens the length of the mid- and forefoot, which decreases the stance time on the foot during gait. An anteriorly located talus shortens the lever arm of the plantar flexors and increases the time needed to step over the foot [9].

Radiographic parameters are the basis for operative decision making in malaligned and painful ankle arthrodesis. Reliable measurement methods are therefore needed to objectively assess the tibio-talar alignment. Several studies focused on the sagittal alignment and tibio-talar offset in the native ankle joint, the arthritic ankle, and in total ankle replacement with regard to component placement [2,7,11,12,13,14,15,16,17,18]. Literature on reliable measurement methods for fused ankles is scarce [3,19,20,21].

The aim of this study was to assess the accuracy and reliability of sagittal tibio-talar alignment measurements in ankle arthrodesis, with focus on the position of the talus to the mechanical longitudinal tibial axis, known as tibio-talar offset.

## 2. Materials and Methods

After approval of the institutional review board (IRB) of the Medical University of Vienna (2002/2013) a retrospective search for patients who underwent ankle arthrodesis between 2006 and 2013 at the Department of Orthopedics (Medical University of Vienna, Austria) has been performed. A manual search through medical records revealed 71 patients. In order to get a homogenous patient population, only patients who underwent isolated tibio-talar arthrodesis using a lateral trans-fibular approach with a two-screw fixation technique, as described by Roger Mann, were included [22]. Patients with differing surgical approaches or fixation techniques (*n* = 6), patients who underwent additional hindfoot arthrodesis (*n* = 15), patients with revision ankle arthrodesis (*n* = 8), and conversion from total ankle arthroplasty to AA (*n* = 5) were excluded. In total, standardized lateral radiographs of 38 fused ankles (20 left, 18 right ankles in 37 patients—17 female, 20 male) who underwent isolated ankle arthrodesis were investigated. All AA were performed by several foot and ankle surgeons. All patients suffered from debilitating end-stage ankle arthritis, including 23 posttraumatic ankles (60.5%), 12 ankles with secondary arthritis (i.e., rheumatoid arthritis, clubfoot sequelae, osteochondral defect, hemophilia, and avascular necrosis of the talus) (31.6%), and three ankles (7.9%) with primary ankle osteoarthritis, respectively. Mean patient age at operation was 53.7 years (range 23–73). Weight bearing radiographs were routinely obtained for radiographic follow-up in a standardized fashion. At our institution, standing weight bearing lateral radiographs were recorded after a minimum of six months postoperatively and were subsequently used for radiographic measurements. Bony union was detectable on x-ray in all ankle fusions. Lateral radiographs were taken with the foot and ankle positioned parallel to the film. The radiographic view comprised the foot and ankle, and the midshaft of the tibia. Radiography has been performed digitally using a SID (source-to-image distance) of 125 cm at 51 kV and 12 mAs (Multix Fusion, Siemens, Erlangen, Germany).

Three independent observers (a foot and ankle surgeon, a senior orthopedic resident, and a final year medical student) measured the sagittal tibio-talar alignment and tibio-talar offset, according to a preset measurement protocol. All images were blinded to patient data and ordered randomly. The measured values were recorded by an independent research assistant and the observers were blinded to results assigned by the other observers. The radiographic assessment was performed twice at a minimum interval of one week between both sessions [21,23]. All radiographic measurements were performed with use of a picture archiving and communication system (PACS) (IMPAX; Agfa HealthCare, Mortsel, Belgium) software.

### 2.1. Radiographic Measurements

For the evaluation of the sagittal tibio-talar angle (STTA), two methods were used. In both methods, the longitudinal tibial axis was determined by drawing a line connecting two points in the middle of the proximal and distal tibial shaft [10,21,24].

The longitudinal axis of the talus was determined by two previously described methods:

Method 1: by drawing a line from the inferior aspect of the posterior tubercle of the talus to the most inferior aspect of the talar neck [1];
Method 2: by drawing a line connecting the midpoints of a line bisecting the talar neck and another line bisecting the talar body [9].

**Figure 1:**
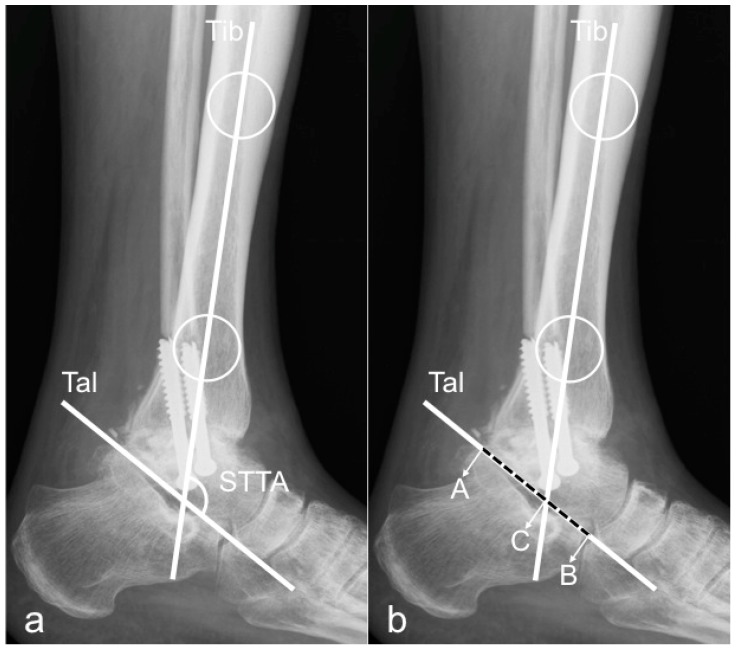
Method 1 for STTA and mTT-ratio measurement. Lateral ankle radiograph demonstrating Method 1: Tib = longitudinal axis of the tibia, created by connecting two points in the middle of the proximal and distal tibial shaft. Tal = axis of the talus, defined by a line drawn from the inferior aspect of the posterior tubercle of the talus to the most inferior aspect of the talar neck. Figure (**a**) depicts measurement of the sagittal tibio-talar angle (STTA). Figure (**b**) shows measurement of the modified tibio-talar (mT-T) ratio.

**Figure 2:**
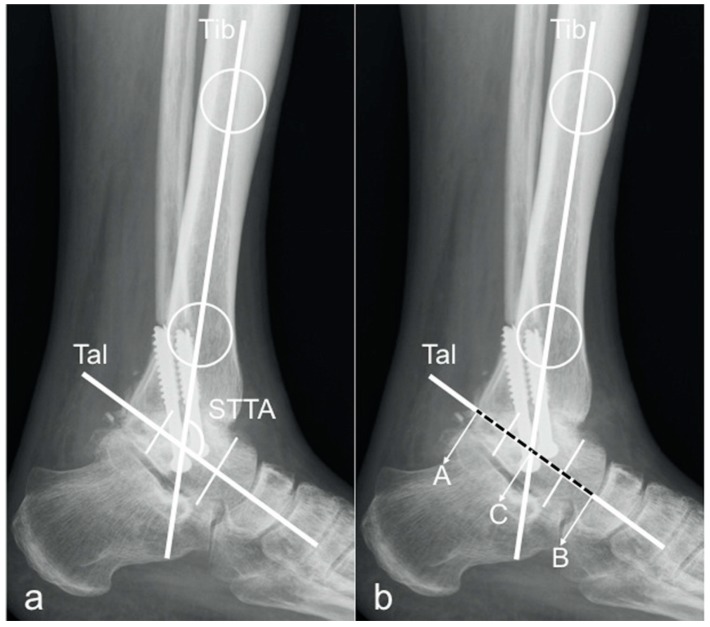
Method 2 for STTA and mTT-ratio measurement. Lateral ankle radiograph demonstrating Method 2: Tib = longitudinal axis of the tibia, created by connecting two points in the middle of the proximal and distal tibial shaft. Tal = or a line drawn connecting the midpoints of a line bisecting the talar neck and another line bisecting the talar body. Figure (**a**) depicts measurement of the sagittal tibio-talar angle (STTA). Figure (**b**) shows measurement of the mT-T ratio.

The angle between these two axes was measured and defined as STTA (Figure 1a and Figure 2a).

Based on these two measurement techniques, the sagittal position of the talus in relation to the tibia (tibio-talar offset) was evaluated by modifying a preexisting technique introduced by Tochigi et al., namely the tibial axis to talus ratio (TT-ratio) [2,14]. The tibio-talar length was measured on the longitudinal talar axis from the posterior talar point (A) to the anterior talar point (B) and the intersection of the talar axis with the longitudinal tibial axis was set as reference point C. The length of the posterior part of the talus (distance AC) and the total length of the talus (distance AB) was then measured and the modified TT-ratio was calculated (Figure 1b and Figure 2b) by the following formula:

modified T-T ratio (%) = (AC/AB) × 100

**Figure 3:**
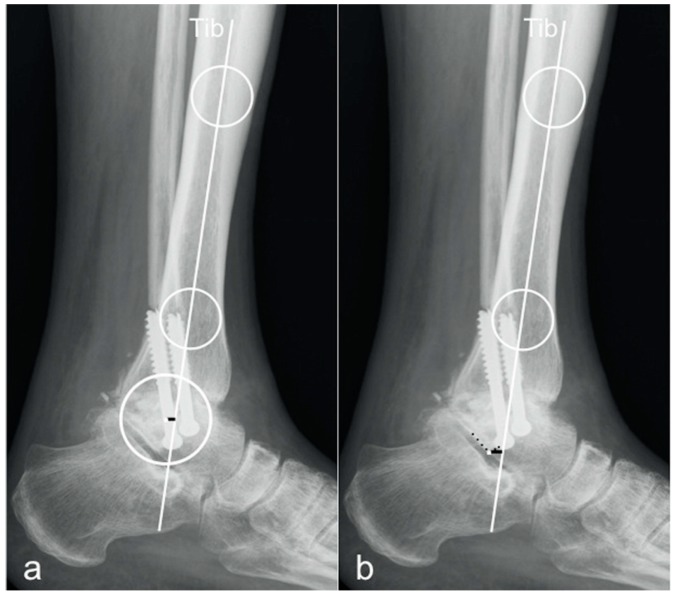
TibCOR and ProcLAT measurement for the tibio-talar offset measurement. Figures are depicting the two different methods for measuring the tibio-talar offset: the distance from the center of a circle manually fitted to the talar dome surface (representing the center of rotation of the ankle joint) and the longitudinal axis of the tibia (**a**), the distance from the tip of the lateral process of the talus to the longitudinal axis of the tibia (**b**).

For measuring the sagittal talar offset by means of an absolute distance (talar center of rotation relative to the longitudinal tibial axis) two different methods described in literature have been found to define the center of rotation of the ankle joint. By Magerkurth et al., a circle was created matching the trochlear curvature of the talar dome and the corresponding center of the circle was determined as center of rotation (tibCOR) [7,10,24]. Paley et al. set the lateral process of the talus as radiographic reference representing the center of rotation of the ankle joint (procLAT) [9]. Both methods were drawn and the corresponding distances (mm) were measured on the radiographs, according to the following definitions (Figure 3):

tibCOR: the distance from the center of a circle manually fitted to the talar dome surface (representing the center of rotation of the ankle joint) and the longitudinal axis of the tibia [7,10,24].
procLAT: the distance from the tip of the lateral process of the talus to the longitudinal axis of the tibia [9].

### 2.2. Assessment of Measurement Reliability, Accuracy, and Agreement

Reliability was defined as consistency of the measurements, and accuracy was defined as proximity of measurements values between the used methods, respectively. Intraobserver reliability was assessed on the basis of the intraclass correlation coefficient (ICC) of each method for each observer and likewise interobserver reliability on the basis of the agreement among the three observers for each method. The first measurement by each observer was used for the analysis of interobserver reliability. The repeated measurements for intraobserver reliability were performed at a mean interval of 1.2 weeks (range 1 to 2 weeks) [9]. Accuracy was compared using the mean differences for angular measurements (STTA) and distance measurements (tibCOR and procLAT). Bland Altman plots were drawn for graphic presentation of differences in interobserver agreement for measuring the tibio-talar offset using tibCOR and procLAT [25].

### 2.3. Statistical Analysis

A sample size analysis was conducted prior to medical records review in order to determine the minimum number of ankle radiographs required to obtain sufficient statistical power. A minimum sample size for reliability evaluation was calculated at 36 ankles by setting the intraclass correlation coefficient (ICC) target as 0.8, 95% confidence interval as 0.2, and number of observers as three with a Bonett’s approximation.

Intraclass correlation coefficient (ICC) and the corresponding 95% confidence intervals were calculated for all continuous variables within and between observers to estimate the intraobserver and interobserver reliability. The ICCs were calculated using a two-way random-effect model, assuming a single measurement and absolute agreement. Intraobserver and interobserver reliability was rated as excellent (>0.90), high (0.70 to 0.89), moderate (0.50 to 0.69), low (0.26 to 0.49), or minimal (correlation coefficient, <0.25).

Accuracy of the STTA measurement methods and the tibio-talar offset measurement methods has been evaluated by using a two-tailed Student t test.

All analyses were performed using SPSS 20.0 for Windows XP (SPSS Inc., Chicago, IL, USA), and the level of significance was set at *p* < 0.05.

## 3. Results

### 3.1. Intraobserver Reliability

Intraobserver reliability for the STTA revealed excellent reliability for method 1 in observer 1 and high reliability in observer 2 and 3, respectively. Reliability for the STTA using method 1 was 0.966 (CI 95% 0.935–0.982) for observer 1, 0.881 (CI 95% 0.77–0.938) for observer 2, and 0.853 (CI 95% 0.698–0.926) for observer 3. Reliability for method 1, mT-T ratio was 0.922 (CI 95% 0.847–0.96) for observer 1, 0.93 (CI 95% 0.859–0.965) for observer 2, and 0.771 (CI 95% 0.556–0.881) for observer 3.

Tibio-talar offset measurement with tibCOR showed excellent intraclass correlation with 0.938 (CI 95% 0.881–0.967) for observer 1 and 0.96 (CI 95% 0.916–0.98) for observer 2 and high reliability with 0.878 (CI 95% 0.758–0.937) for observer 3. All ICCs are shown in Table 1.

### 3.2. Interobserver Reliability

Method 1 showed excellent interobserver reliability for measurement of the STTA and the mT-T ratio, whereas interobserver reliability was classified high for method 2. The interobserver coefficient (ICC) of repeatability for the STTA was 0.924 (CI 95% 0.862–0.959) for method 1 and 0.849 (CI 95% 0.726–0.919) for method 2. The ICC for the mT-T ratio was 0.836 (CI 95% 0.721–0.909) for method 1 and 0.731 (CI 95% 0.542–0.851) for method 2. Method 1 yielded the highest interobserver reliability for both STTA and the mT-T ratio. Interobserver reliability for tibio-talar offset distance was 0.89 (CI 95% 0.812–0.939) for tibCOR and 0.878 (CI 95% 0.791–0.932) for procLAT, displaying superior results for tibCOR (Table 1).

### 3.3. Accuracy

Angular measurements of the sagittal tibio-talar alignment are outlined in Table 2.

The mean difference between the STTA measured by method 1 and method 2 was 1.25 degrees (CI 95% −0.46–2.96). The difference between the measurement methods was not statistically significant (*p* = 0.154). Concerning tibio-talar offset measurements, we found a significant difference between the tibCOR and the procLAT method (mean difference −2.01 mm; CI 95% −3.11–0.91; *p* < 0.000) (Table 2).

### 3.4. Agreement

**Figure 4:**
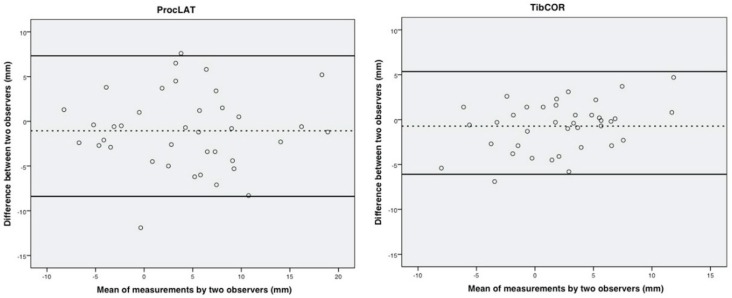
Bland Altman Plots. Bland and Altman plots depicting the procLAT and tibCOR measurements by two observers, demonstrating better agreement with use the tibCOR (right) compared with use of the procLAT (left). The average of the measurements made by two observers is plotted against the difference between the measurements made by those two observers. The dashed lines represent the mean value of all differences between the two observers, and the dotted lines represent the 95% limits of agreement.

Bland Altman plots were drawn for comparison of the tibCOR and procLAT measurements depicting better agreement with use of tibCOR compared with use of procLAT (Figure 4).

## 4. Discussion

Optimal alignment in the frontal and sagittal plane of AA is essential for successful clinical long-term outcome [1,3,5,6,26]. Malalignment following tibio-talar fusion is recognized to be an underlying cause of persisting chronic pain in the hindfoot [3,5,27,28]. Plantarflexion malalignment decreases the sagittal motion of the foot and can lead to genu recurvatum [1,5,29], varus malalignment shifts the loading axis on the lateral side of the foot [1]. These adverse biomechanical conditions lead to abnormal gait patterns and to increased hindfoot symptoms [3,5,26,27]. Establishing accurate and reproducible radiographic measurement methods for the sagittal tibio-talar angle and offset in ankle arthrodesis is essential for evaluating the reconstruction of anatomical tibio-talar joint alignment. Various studies have examined ankle alignment in both native ankles, and ankles after total ankle replacement [2,10,12,14,24,30,31,32]. To date, there exists no standardized radiographical method to evaluate sagittal malalignment in ankle arthrodesis. In the present study, the inter- and intraobserver reliability of different proposed radiographic measurement methods for the sagittal tibio-talar angle (STTA) and the tibio-talar offset have been investigated for the first time. Additionally, measurement accuracy and interobserver agreement was assessed. Furthermore, we were able to assess the most reliable methods applicable in further clinical research.

Two different methods to measure the STTA and the modified T-T ratio were compared. Method 1 used a line drawn from the inferior aspect of the posterior tubercle of the talus to the most inferior aspect of the talar neck [1] to determine the longitudinal talar axis. Method 1 yielded the highest interobserver reliability for both STTA and the mT-T ratio. For the STTA excellent interobserver, reliability was achieved for method 1 (0.924; CI 95% 0.862–0.959) as well as excellent intraobserver reliability in observer 1 (0.966; CI 95% 0.935–0.982), and high intraobserver reliability in observers 2 and 3, respectively. For assessment of the modified T-T ratio, the interobserver reliability for method 1 was high (0.836; CI 95% 0.721–0.909), and intraobserver reliability was excellent for observer 1 and 2, and high for observer 3. For measuring the tibio-talar offset in AA, two methods differing by definition of the center of rotation of the ankle joint were used and consecutively compared [7,9,10,24]. Interobserver reliability displayed better results for tibCOR (ICC 0.89; CI 95% 0.812–0.939) and revealed excellent intraobserver measurement reliability for observer 1 (ICC 0.938; CI 95% 0.881–0.967), observer 2 (0.96; CI 95% 0.916–0.98), and high reliability for observer 3 (0.878; CI 95% 0.758–0.937).

The mean difference between both measurement methods for the STTA was not statistically significant (*p* = 0.154). Intergroup analysis comparing the tibCOR and the procLAT measurements methods revealed statistically significant differences of the mean (*p* < 0.000).

This retrospective study is related to several limitations. Prior to patient charts review, a sample size analysis was conducted in order to determine the minimum number of ankles required to obtain sufficient statistical power. With a cohort of 38, on the one hand this study had adequate statistical power to determine reliable measurements, on the other hand, only a relatively small number of ankles has been included. Another limiting fact is that only postoperative radiographs were analyzed. Therefore, no measurement of the initial pre-operative ankle alignment and sagittal tibio-talar offset could be analyzed, neither could a comparison be carried out. Although radiographic analysis methods have already been established in both native ankles and ankles replaced by total ankle prosthesis [2,10,14], some proposed landmarks in fused ankles are difficult to assess. Some alterations had to be performed to apply these methods accordingly. Paley described a method to determine the longitudinal talar axis by drawing a line connecting the midpoints of a line bisecting the talar neck and a second line bisecting the talar body [9]. Due to the performed fusion of the tibio-talar joint, it was necessary to adopt the above described technique to the present conditions (i.e., in cases of major bone loss at the talar dome it was somehow difficult to bisect the talar body). This subjective finding was reflected in reliability analysis outlining lower ICC levels for the method advocated by Paley et al. (Method 2). A further limitation was that scaled radiographs were not available for this patient cohort. Taking this potential drawback into consideration, we were not able to compare our results from AA to the already published normal values for tibio-talar offset in native ankles by Magerkurth et al. [24]. Nevertheless, the used radiographs were taken in a standardized set-up using commonly available radiographic measurement tools. To attenuate for this limitation, we measured the sagittal alignment using the modified tibio-talar ratio and therefore establishing a reproducible measuring device. Furthermore, although a standardized protocol was being used, there are still some technical limitations. The radiographic projection can be affected by position and rotation of the ankle during standing radiograph as well as by changing position of the radiographic beam [2,12,14].

Measurement of the distance from the talar center of rotation to the longitudinal tibial axis defined as tibio-talar offset was introduced by Magerkurth et al. in a cohort of healthy (native) ankles using the tibCOR method. They reported a mean tibCOR of 1.7 mm (SD = 2.1 mm) in term of a slight anterior offset [24]. The majority of our AA also showed an anterior position of the talus in relation to the tibia (mean tibCOR: 1.96 mm; SD = 4.54). TibCOR measurements showed more reproducible results compared to procLAT. The lateral talar process as landmark is inadequate if the transverse ankle position is not perfectly controlled [2,14]. Our results underlined the findings by Tochigi et al. [2,14].

Tochigi et al. proposed different ratios for offset measurement and revealed the TT-ratio to be the most reproducible method in arthritic ankles [2,14]. We found that a modification of the TT-ratio by using the axis of the talus instead of the proposed talar reference line. This is a line parallel to the floor drawn through the posterior talar point (identified as the intersection between the contours of the posterior subtalar articular surface and the postero-superior calcaneal cortex), simplified the measurement substantially, and implemented the measurement of the sagittal tibio-talar angle at the same time.

## 5. Conclusions

In conclusion, the present study performed reliability analysis for sagittal tibio-talar alignment measurements in ankle arthrodesis for the first time. Summarizing reliability, accuracy, and agreement results, the STTA should be measured by defining the longitudinal axis of the talus as a line drawn from the inferior aspect of the posterior tubercle of the talus to the most inferior aspect of the talar neck (Method 1). A modified T-T ratio also showed high interobserver reliability. The proposed tibCOR and procLAT methods for tibio-talar offset evaluation were subsequently compared. According to our results, the use of the tibCOR method for evaluation of the tibio-talar offset can be recommended for future scientific radiographic measurements in AA. This method measures the distance from the center of a circle manually fitted to the talar dome surface to the longitudinal axis of the tibia. Establishing standardized measurement methods for alignment analysis allows a plausible comparison and evaluation of radiographic outcome in different patient cohorts. In addition, the surgical approach and exposure might have an influence on the intraoperative alignment control and could consecutively affect the final alignment. Future studies should also evaluate intraoperative alignment measurement techniques, which might be a useful tool. Based on the findings of our study, criteria for postoperative radiographic malalignment in ankle arthrodesis and subsequent clinical and functional consequences on the outcome could be established in further studies.

## Figures and Tables

**Table 1:** Intra- and interobserver reliability.

**ICC INTRAOBSERVER RELIABILITY**						
	**STTA Method 1**	**95% CI lower**	**upper**	**STTA Method 2**	**95% CI lower**	**upper**
Observer 1	0.966	0.935	0.982	0.852	0.687	0.927
Observer 2	0.881	0.77	0.938	0.892	0.793	0.944
Observer 3	0.853	0.698	0.926	0.832	0.64	0.917
	**mT-T ratio Method 1**	**95% CI lower**	**upper**	**mT-T ratio Method 2**	**95% CI lower**	**upper**
Observer 1	0.922	0.847	0.96	0.768	0.515	0.885
Observer 2	0.93	0.859	0.965	0.846	0.703	0.92
Observer 3	0.771	0.556	0.881	0.83	0.675	0.911
	**tibCOR**	**95% CI lower**	**upper**	**procLAT**	**95% CI lower**	**upper**
Observer 1	0.938	0.881	0.967	0.937	0.879	0.967
Observer 2	0.96	0.916	0.98	0.981	0.96	0.991
Observer 3	0.878	0.758	0.937	0.83	0.673	0.912
**ICC INTEROBSERVER RELIABILITY**						
	**STTA Method 1**	**95% CI lower**	**upper**	**STTA Method 2**	**95% CI lower**	**upper**
	0.924	0.862	0.959	0.849	0.726	0.919
	**mT-T ratio Method 1**	**95% CI lower**	**upper**	**mT-T ratio Method 2**	**95% CI lower**	**upper**
	0.836	0.721	0.909	0.731	0.542	0.851
	**tibCOR**	**95% CI lower**	**upper**	**procLAT**	**95% CI lower**	**upper**
	0.89	0.812	0.939	0.878	0.791	0.932

This table shows the intra- and interobserver reliability for both the sagittal tibio-talar angle (STTA) and the tibio-talar ratio (mT-T ratio) as well as the tibio-talar offset by distance measurements (tibCOR and procLAT). Intraclass Correlation Coefficients (ICC) with 95% confidence intervals (95% CI) are outlined. The values are given separately according to the two methods for drawing the longitudinal axis of the talus as described in the main text. The highest ICCs for inter- and interobserver reliability are marked green.

**Table 2:** Radiographic measurements.

	Mean	Range	SD	95% CI Lower	Upper	*p*-Value
STTA						
Method 1	109.11°	78.1–131.8	8.54	107.99	110.22	
Method 2	107.86°	72.8–129.7	10	106.56	109.17	
All	108.49°	72.8–131.8	9.31	107.63	109.34	
difference	1.25°			0.46	2.96	0.154
mT-T ratio						
Method 1	46.53%	9.82–141.83	14.1	44.69	48.37	
Method 2	31.59%	7.99–70.23	11.02	30.15	33.03	
all	39.06%	7.99–141.83	14.69	37.7	40.41	

tibCOR	1.96 mm	−10.7–16.9	4.54	1.36	2.55	
procLAT	3.97 mm	−10.6–20.9	7.13	3.04	4.9	
difference	−2.01 mm			−3.11	0.91	<0.000

This table shows mean values with standard deviations (SD) and 95% confidence intervals (95% CI) for the STTA, mT-T ratio for both methods and tibCOR and procLAT measurements. The green box indicates a statistically significant difference.
